# An Adaptive STDP Learning Rule for Neuromorphic Systems

**DOI:** 10.3389/fnins.2021.741116

**Published:** 2021-09-24

**Authors:** Ashish Gautam, Takashi Kohno

**Affiliations:** ^1^Department of Electrical Engineering and Information Systems, Graduate School of Engineering, The University of Tokyo, Tokyo, Japan; ^2^Institute of Industrial Science, The University of Tokyo, Tokyo, Japan

**Keywords:** adaptive STDP, synaptic weight resolution, neuromorphic hardware, pattern detection, neuromorphic computing, biomimetic silicon neuron, silicon synapse

## Abstract

The promise of neuromorphic computing to develop ultra-low-power intelligent devices lies in its ability to localize information processing and memory storage in synaptic circuits much like the synapses in the brain. Spiking neural networks modeled using high-resolution synapses and armed with local unsupervised learning rules like spike time-dependent plasticity (STDP) have shown promising results in tasks such as pattern detection and image classification. However, designing and implementing a conventional, multibit STDP circuit becomes complex both in terms of the circuitry and the required silicon area. In this work, we introduce a modified and hardware-friendly STDP learning (named adaptive STDP) implemented using just 4-bit synapses. We demonstrate the capability of this learning rule in a pattern recognition task, in which a neuron learns to recognize a specific spike pattern embedded within noisy inhomogeneous Poisson spikes. Our results demonstrate that the performance of the proposed learning rule (94% using just 4-bit synapses) is similar to the conventional STDP learning (96% using 64-bit floating-point precision). The models used in this study are ideal ones for a CMOS neuromorphic circuit with analog soma and synapse circuits and mixed-signal learning circuits. The learning circuit stores the synaptic weight in a 4-bit digital memory that is updated asynchronously. In circuit simulation with Taiwan Semiconductor Manufacturing Company (TSMC) 250 nm CMOS process design kit (PDK), the static power consumption of a single synapse and the energy per spike (to generate a synaptic current of amplitude 15 pA and time constant 3 ms) are less than 2 pW and 200 fJ, respectively. The static power consumption of the learning circuit is less than 135 pW, and the energy to process a pair of pre- and postsynaptic spikes corresponding to a single learning step is less than 235 pJ. A single 4-bit synapse (capable of being configured as excitatory, inhibitory, or shunting inhibitory) along with its learning circuitry and digital memory occupies around 17,250 μm^2^ of silicon area.

## Introduction

The primary goal of neuromorphic computing since its inception in the late 1980s has been to design low-power electronic circuits that can mimic human cognition as well as shed light on the complex mechanisms underlying neural computation. Toward this endeavor, research groups across the globe have pursued different design methodologies. Inspired by the architecture of the brain, the common feature in all the approaches is the core components of a neuromorphic system consisting of neuronal soma and synaptic circuits empowered with learning mechanisms—supervised or unsupervised. These circuits are designed based on the mathematical models of the cell membrane, ionic dynamics in the cell, and synapses, which in turn are constructed from electrophysiological data measured from them. The learning mechanism is also the most important component in the neuromorphic systems. Spike time-dependent plasticity (STDP) is the most well-known learning rule for unsupervised learning in the brain, which is implemented in many neuromorphic systems. The STDP learning updates the synaptic weight of a particular synapse based on the interspike interval between the spikes of the pre- and the postsynaptic neurons. STDP-based unsupervised learning has been successful in tasks such as pattern detection (Masquelier et al., 2008, 2009) and image classification (Diehl and Cook, 2015), achieving high performance in simulation.

Depending on the implementation details and the capability of the models chosen, neuromorphic systems can be categorized as bioinspired (simpler models, easier to implement) (Vogelstein et al., 2007; Merolla et al., 2014; Davies et al., 2018), biomimetic (detailed models, take up relatively more area and power) (Nease et al., 2012; Schemmel et al., 2017), or an optimized combination of both (Benjamin et al., 2014; Qiao et al., 2015). The choice of the model is driven by the desired application and involves making a trade-off between power consumption, area, and ease of design. The complementary metal-oxide-semiconductor (CMOS) circuit is generally used in low-power neuromorphic circuits. The integration of novel non-volatile memory devices (e.g., memristors and ferroelectric field-effect transistors) for learning rule implementation is a trend these days, but conventional CMOS circuit will be a realistic solution from the viewpoint of cost, stability, and integration until these devices get matured.

Spike time-dependent plasticity learning models that have achieved high performance in simulation (Masquelier et al., 2008, 2009; Diehl and Cook, 2015) use the entire 64-bit floating-point operation available on standard digital computers. Implementing the STDP learning with high-resolution weights (even 8 to 10 bits) in CMOS digital memory circuit requires a large number of transistors which leads to a big footprint and high-power consumption. Because non-volatile memory devices are still in their prototype stage, we propose a modified bioinspired learning rule, adaptive STDP learning, which can achieve good performance with lower resolution memory.

The long-term goal of our research is to develop a biologically plausible neuromorphic system that can be used in biohybrid systems such as the brain–machine interface (BMI) decoder (Boi et al., 2016). Toward this goal and to demonstrate the capability of this learning rule, we choose a spike pattern detection model presented in Masquelier et al. (2008) that takes into account the background activity of presynaptic neurons. In this model, repeatedly appearing spike patterns hidden in the spontaneous background firing activity are detected by the STDP learning rule. In this work, we apply the proposed adaptive STDP learning rule in this pattern recognition task and present the simulation results. The key idea behind the adaptive STDP learning is that the loss in performance generally accompanied by the use of low-resolution synapses can be compensated by adapting the parameter controlling the long-term depression function (in the STDP learning) over the course of training. The circuit controlling this adaptation can be shared with all the synapses involved in the task; thus, the overhead is minimal. In addition, the bit update (modification in the value of synaptic weight) at any instant in time is restricted to just 1 bit, which simplifies the circuitry considerably. Our results show that the presented learning rule (using just 4-bit weight) is equally powerful as the high-resolution STDP learning. Along with these results obtained using simulation of ideal models in python, we also present the circuits that implement the neuron and synapse models used in this work to indicate that these models are suitable for neuromorphic implementation. The manuscript is organized as follows: the next section comprises the details of the pattern recognition task, the adaptive STDP learning, and the results. This is followed by a discussion and conclusion.

## Materials and Methods

We begin by describing the setups used for the pattern recognition task. Then the adaptive STDP learning rule is introduced. This is followed by a description of the neuron and synapse models used in the task along with the schematic of the corresponding circuit modules.

### Pattern Recognition Setup

Repetitive spike patterns precise to the scale of milliseconds and spontaneous background firing activity with a fano factor in the range of 1 to 1.2 are ubiquitous in the cortex (Koch, 1999). The task of pattern recognition here involves detecting these spike patterns hidden within the spontaneous background firing activity. In Masquelier et al. (2008), it was demonstrated that a single neuron equipped with STDP learning could perform this task of detecting one hidden pattern within spontaneous background firing with a success rate of 96%. The random background firing activity is modeled by an inhomogeneous Poisson process, and the single spike pattern to be detected is hidden at irregular intervals. Like the setup presented in Masquelier et al. (2008), our setup comprises a neuron receiving spikes from *N*_*aff*_ inputs through excitatory synapses. These synapses are activated by spike trains, each generated independently using an inhomogeneous Poisson process with a variable instantaneous firing rate ranging between 0 and 90 Hz. The maximum possible rate of change was chosen so that spiking frequency could go from 0 to 90 Hz in 50 ms. The setup also made sure that each afferent spikes at least once within a 50-ms duration, making the minimum spiking frequency 20 Hz. After the generation of random spike trains, a part of it covering 50 ms duration is randomly chosen and copied; this is the pattern to be detected. The original spike train is then discretized into 50 ms sections, and randomly, one of this section is chosen and replaced by the copied spike train. Then based on the desired pattern repetition frequency (chosen to be 25% or 10% in our simulation setups), a certain number of these sections are randomly chosen from the original spike train and replaced by the pattern to be detected. Consecutive 50 ms sections are avoided in this process. The population average firing rate of these afferents in 10 ms time bins is approximately the same throughout the input spike train, making sure that the 50-ms sections comprising spike patterns have the same population average spike rate as the rest of the input spike train [10 ms time bins was chosen because it was the time constant of the LIF neuron used in Masquelier et al. (2008) and we also use the same value]. This population average firing rate is around 54 Hz. Then to make the pattern recognition task extra difficult, additional spontaneous 10 Hz noise is added to all spike trains along with a random jitter on the precise spike time of the spike patterns to be detected. This additional noise overlaps with the spike patterns, and the population average firing rate in 10 ms bins with this addition is around 64 Hz. Without this additional noise and jitter, all the afferents fire in precisely the same manner at every pattern presentation. The jitter added to the spike pattern is modeled as a Gaussian random variable with a mean zero and standard deviation of 1 ms.

In the reference simulation setup in Masquelier et al. (2008), there are 2,000 afferents, of which 1,000 of the afferents receive repeated spike patterns hidden in the background spiking activity as described above, and the remaining 1,000 afferents receive stochastic spike trains that model the background firing activity (with no repeated hidden patterns). In our work, we perform simulations using three different setups. Setup 1 has 2,048 afferents, of which stochastic spike trains containing hidden patterns are received by 1,024 afferents, and the remaining 1,024 afferents receive stochastic spike trains that model the background firing activity (they do not contain any repeated hidden patterns). It corresponds to the reference setup in Masquelier et al. (2008). In setup 2, we choose the number of afferents to be 1,024, and in setup 3, the number of afferents is reduced further to 256. In setups 2 and 3, all the afferents receive stochastic spike trains with patterns hidden within. In the setup with 1,024 afferents, we use spike patterns described above, the same as in setup 1 and (Masquelier et al., 2008). In the setup with only 256 afferents, the additional 10-Hz spontaneous noise and jitter in the spike times within the pattern are not included. Everything else remains the same. It is empirically known that the performance of the pattern recognition task degrades with a reduction in the number of afferents. The additional noise and jitter are removed from setup 3 to “normalize” the difficulty of the task, as the number of active afferents is reduced to one-fourth of the original value. Setup 1 compares the performance of the adaptive STDP learning with the STDP learning with 64-bit floating-point operation. The other two setups are for neuromorphic applications with light-weighted neuromorphic circuits. Because of limited chip area, the number of synapses in a neuromorphic chip is generally restricted; hence, we reduce the number of afferents in our setup too.

The spike trains with patterns to be detected buried in the background activity are generated for 225 s. We executed 100 runs for each simulation setup, and each run is 450 s long. Because of memory constraints, the 225-s-long input is repeated twice to get 450 s-long input. Because the period of 225 s is sufficiently longer than the 50-ms time bin, this repetition is expected to have very few additional effects on the learning process. In the simulation setup presented in Masquelier et al. (2008), 150-s-long input was repeated thrice to get 450-s-long input spike train. A summary of the experimental setups is given in Table 1.

**TABLE 1:** Experimental setups for pattern recognition.

	**Setup in** Masquelier et al. (2008)	**This work setup 1**	**This work setup 2**	**This work setup 3**
Number of afferents (*N*_aff_)	2,000	2,048	1,024	256
Number of active afferents	1,000	1,024	1,024	256
Stochastic spike trains with hidden patterns modeled by	Inhomogeneous Poisson process and 10 Hz spontaneous noise and jitter	Inhomogeneous Poisson process and 10 Hz spontaneous noise and jitter	Inhomogeneous Poisson process and 10 Hz spontaneous noise and jitter	Inhomogeneous Poisson process
Population average spike rate in 10 ms time bins	64 Hz	64 Hz	64 Hz	54 Hz

### Adaptive STDP Learning

**FIGURE 1:**
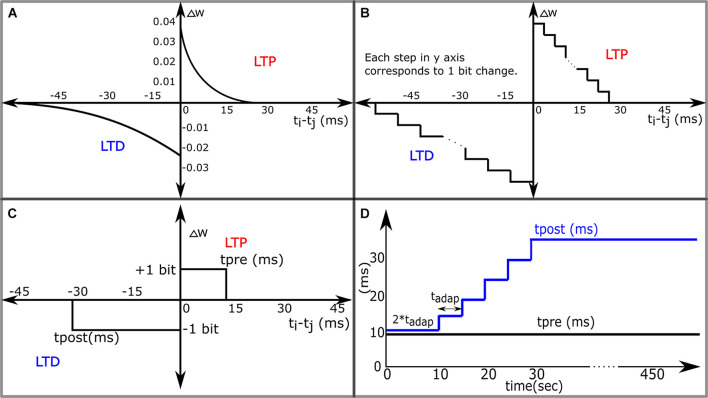
Variants of the STDP learning rules. **(A)** The STDP learning rule. **(B)** The STDP Learning Rule for Neuromorphic Systems with DACs and digital memories configuring synaptic weights. **(C)** 1-bit rectangular STDP. **(D)** Adaptive STDP: the value of tpost in 1-bit rectangular STDP is varied in steps over time.


When using the STDP learning, the network dynamics evolve as follows. The spike inputs received *via* synapses cause the neuron to spike. If the input spike (presynaptic spike) activating a synapse arrives before the postsynaptic spike (if the synapse contributes to the spiking of the neuron), the value of its synaptic weight is potentiated. On the other hand, if the input spike (presynaptic spike) activating the synapse arrives after the postsynaptic spike (if the synapse does not contribute to the spiking of the neuron), the value of its synaptic weight is depressed. The closer the pre- and the postsynaptic spikes are to each other, the higher is the value of potentiation or depression. The update made in the value of synaptic weight decays exponentially with increasing interspike intervals, as shown in Figure 1A. Mathematically, this is represented as:

(1) $$△\,w_{j}=\{\begin{array}{c} a^{+}\cdot \exp\left(\frac{t_{j}-t_{i}}{\tau^{+}}\right)\,\text{if}\,t_{j}\leq t_{i}\,\left(\text{LTP}\right),\\ a^{-}\cdot \exp\left(-\frac{t_{j}-t_{i}}{\tau^{-}}\right)\,\text{if}\,t_{j}>t_{i}\,\left(\text{LTD}\right). \end{array}$$

Here, *t_j* is the timing of the presynaptic spike and *t_i* is the timing of the postsynaptic spike. τ^+^(τ^−^) is the time constant of the decaying exponential controlling potentiation (depression) of synapses, and *a*^+^ (*a*^−^) is the learning rate that controls the maximum change in the value of synaptic weight while in the potentiation (depression) process. The product *a*^+^τ^+^ is generally set smaller than the product *a*^−^τ^−^. In the pattern recognition task using STDP learning, with the onset of input spikes, the neuron receives current *via N*_*aff*_ synapses, each initialized with the same value of synaptic weight. In the beginning, the postsynaptic spikes arise in response to random coincidence in the input spikes. The initial value of synaptic weights is set so that during the initial phase of the run, the spiking frequency is relatively high (50–160 Hz), and the postsynaptic spikes occur both inside and outside the pattern. While the pattern to be detected does not appear and the presynaptic spike train comprises only random spikes, all the synapses get depressed over time. This happens because *a*^+^τ^+^<*a*^−^τ^−^ and depression dominates over potentiation. However, in the presence of spike patterns repeating at irregular intervals, the synapses associated with the spike pattern are activated much more than the other synapses receiving random spikes. As a result of this over the course of learning, a relationship emerges between pre- and postsynaptic spikes. The synapses associated with the spike pattern get potentiated, while the remaining synapses get depressed, and therefore, the neuron spikes in response only to the spike pattern. As the pattern is 50 ms long and the time constant of the neuron used in Masquelier et al. (2008) was 10 ms, the parts of the pattern that the neuron responds to are determined by chance initially, but while learning, the timing of the spike within the pattern decreases and the neuron gets to spike very close to the beginning of the pattern.

The success rate of the pattern recognition task described above relies on the resolution of synaptic weights, the minimum allowable change in the value of synaptic weight either during potentiation or depression. The smaller the change (or the higher the resolution), the higher is the success rate, because with a small change in the value of synaptic weights, the total synaptic current depolarizing the neuron does not change substantially in a small duration even if many synapses are updated simultaneously, this keeps the learning process stable, and the neuron does not stop spiking due to a sudden depression of synapses. In the pattern detection task presented in Masquelier et al. (2008), the synaptic resolution is quite high using the entire 64-bit floating-point precision. Designing a neuromorphic system with relatively high-resolution weight (∼8- to 10-bit fixed-point) takes a significant amount of silicon area as well as power. In such systems (Vogelstein et al., 2007; Cassidy et al., 2011; Pfeil et al., 2012; Moradi and Indiveri, 2013), the STDP circuit is generally designed by implementing a function approximating the exponential dependence of LTP and LTD as shown in Figure 1B. However, even designing such a circuit gets very complicated involving the use of modules like adders, subtractors, lookup tables or comparators, and digital to analog converters. The circuit design of the STDP circuit can be simplified if the change in the value of synaptic weight is restricted to 1 bit at any instant of time. This can be achieved by modification of the STDP learning rule to 1-bit rectangular STDP learning rule as shown in Figure 1C and mathematically expressed as:


(2) $$△\,w_{j}=\left\{ \begin{array}{c} +1\,\mathrm{bit},\mathrm{if}\,t_{j}\leq t_{i}\,\text{and}\,t_{i}-t_{j}<t\,\text{pre}\,\left(\text{LTP}\right),\\ -1\,\mathrm{bit},\mathrm{if}\,t_{j}>t_{i}\,\mathrm{and}\,t_{j}-t_{i}<t\,\text{post}\,\left(\text{LTD}\right), \end{array}\right.$$

where, *t*_*pre*_ is the maximum delay of the postsynaptic spike after the presynaptic spike that leads to potentiation (LTP), and *t*_*post*_ is the maximum delay of the presynaptic spike after the postsynaptic spike that leads to depression (LTD). However, in our simulations, we observed that using this learning rule with low-resolution synapses (e.g., 4 bits) leads to poor performance in the pattern recognition task. In Cassidy et al. (2007, 2011), low complexity synthetic implementation of STDP was explored using basic combinational digital logic gates. They implemented the STDP functions shown in Figures 1A–C and a few others and evaluated their performance using a balanced excitation experiment, based on the experiment run by Song et al. (2000). The experiment involves exciting a LIF neuron *via* STDP-enabled synapses activated by independent Poisson spikes. The judgment criterion was based on the shape of the bimodal distribution of synaptic weights obtained after learning. Results obtained from the STDP function in Figure 1A were used as the baseline condition for evaluation. They observed that the STDP function in Figure 1B performed best, and reasonable bimodal distributions were obtained from the function in Figure 1C by manual tuning of parameters *t*_*pre*_ and *t*_*post*_. One hundred and twenty-eight 8-bit synapses were used in this study. Though a bimodal distribution is obtained, designing 8-bit synapses is still a major overhead in terms of silicon area and power consumption (in CMOS-based analog circuits, it would occupy 16 times more area than a 4-bit synapse). In addition, in the spike pattern recognition task like the one discussed in this study, obtaining a bimodal distribution of synaptic weights alone is not a criterion for success, because such a distribution is obtained even in the presence of false alarms (neuron spikes outside the 50-ms patterns). The task is considered successful if after learning the neuron spikes within the 50-ms duration where the pattern is present and nowhere outside the pattern.

The degradation in performance with the use of a low-resolution synapse occurs due to the following reason. Because the minimum change in the synaptic weight is not sufficiently small, a single learning step causes a big and sudden change in the synaptic current. Moreover, as the value of *t*_*post*_ is generally larger than *t*_*pre*_, it tends to induce a strong depression of the synapses even those associated with spike patterns at an earlier stage in the learning process and this causes the neuron to stop spiking. Essentially, the neuron gives up before the synapses have a chance to be potentiated by the spike patterns to be detected. On the other hand, if *t*_*post*_ is kept slightly higher than *t*_*pre*_, then the neuron does not stop spiking and many synapses even those not associated with the pattern get potentiated, leading to many false alarms.

To overcome this problem and improve the performance, we propose to modify *t*_*post*_ in steps during the learning phase as shown in Figure 1D. At the beginning *t*_*post*_ is closer to *t*_*pre*_, this gives ample time for the synapses involved in the pattern to be potentiated, along with many extra synapses, and as time progresses, *t*_*post*_ is adapted to higher values leading to depression of synapses not associated with the pattern. *t*_*pre*_ is kept constant and it keeps reinforcing the synapses associated with the spike pattern repeatedly presented. The initial value of synaptic weights is set so that the spiking frequency of the neuron during the initial phase of learning is quite high (50–160 Hz), and as time passes, the neuron becomes selective to spike inputs from the synapses associated with the spike patterns and spikes only when the spike pattern is present. Just like in the case of the STDP rule, chance determines which part of the spike pattern the neuron responds to initially, but over time, the latency to spike within the pattern decreases. Then the neuron learns to spike close to the beginning of the pattern presentation. The weight update is governed by only the most recent pair of pre- and postsynaptic spikes as in the preceding work (Masquelier et al., 2008).

### Neuron and Synapse Model

**FIGURE 2:**
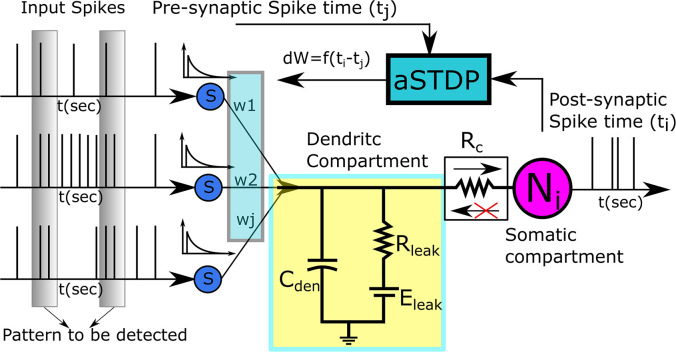
Pattern Recognition Setup. The dendritic compartment is passive and is represented by a dendritic capacitance *C_*den*_*, and a leak resistor *R*_*leak*_. The Adaptive STDP modifies the value of synaptic weights based on the time of the pre- and the postsynaptic spikes.

To design a biologically plausible system that can be used in biohybrid applications such as BMI decoders, a detailed neuron model might be preferable, but designing such a model is not very efficient in terms of silicon area and power consumption. Hence, a trade-off needs to be made between neuro-mimicry and compact low-power circuitry. Toward this goal, we use the reduced compartmental modeling technique (Herz et al., 2006) to model our neuron. It comprises two compartments: a somatic compartment and a dendritic compartment. The somatic compartment is modeled using a biomimetic qualitative neuron model (Kohno and Aihara, 2016; Kohno et al., 2016), and the passive dendritic compartment is modeled using a leak resistor (*R*_*leak*_) and a dendritic capacitor (*C*_den_). All *N*_*aff*_ excitatory synapses connect to the dendritic compartment. The somatic and dendritic compartments are connected *via* a unidirectional resistor that either sources or sinks current into the somatic compartment depending on the membrane potential difference between the dendritic and the somatic compartment. As the name implies, no current flows into or out of the dendritic compartment *via* the unidirectional resistor. The unidirectional resistor is to be implemented using a single-stage transconductance circuit that consumes very low static power in the picowatts range. In terms of its complexity, this neuron model lies in between the point neuron model (where synapses connect directly to the soma) and the two-compartment neuron model (where somatic and dendritic compartments are connected *via* a resistor). Implementing a point neuron model might seem a simpler choice, but its implementation with a biomimetic somatic compartment and a large number of synaptic circuits requires a current conveyor circuit (Chaisricharoen et al., 2010) as an interface module between the soma and the synapses. Without the interface module, the large parasitic capacitance and the leakage current of the synapses affect the membrane capacitance of the soma and disturb the spiking behavior of the neuron. However, to convey the current faithfully, maintaining the precise shape and timing of the synaptic current, the current conveyor needs to be operated using a high bias voltage leading to a significantly high static power consumption in the nanowatts range, whereas the implemented unidirectional resistor consumes power in the picowatts range. Hence, the two-compartment model with a unidirectional resistor was chosen. Figure 2 shows the block diagram of the pattern recognition setup.

**FIGURE 3:**
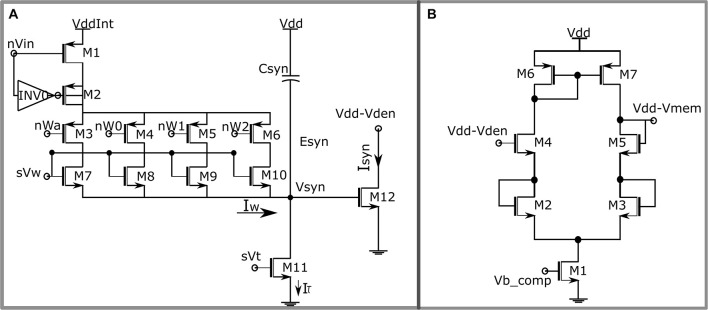
**(A)** Circuit diagram of the synaptic circuit. Dimensions of the transistors: M7 = 0.3758 * (w/l), M8 = w/l, M9 = 2 * (w/l) and M10 = 4 * (w/l) with w = 2 μm, l = 500 nm. **(B)** Single-stage source degenerated transconductance amplifier configured as unidirectional resistor.


The somatic compartment modeled using a biomimetic qualitative neuron model (Kohno and Aihara, 2016) is described by the following equations


(3) $$C_{v}\,\frac{d\,v}{d\,t}=f_{v}\,\left(v\right)-g_{v}\,\left(v\right)+I_{a\,v}-r_{n}\,\left(n\right)-r_{q}\,\left(q\right)+\frac{v_{d\,e\,n}-v}{R_{C}},$$

(4) $$C_{n}\,\frac{d\,n}{d\,t}=f_{n}\,\left(v\right)-g_{n}\,\left(v\right)+I_{a\,n}-r_{n}\,\left(n\right),$$

(5) $$C_{q}\,\frac{d\,q}{d\,t}=f_{q}\,\left(v\right)+I_{a\,q}-r_{q}\,\left(q\right),$$

where, *v*, *n*, and *q* represent the membrane potential, the fast dynamics, and the slow dynamics of the somatic compartment, respectively. *I*_*av*_,*I*_*an*_, and*I_aq_* are constant current parameters.*C_v_*,*C_n_*, and *C_q* are 0.6, 0.9, and 24 pF, respectively. *R_C* is the unidirectional resistor, which is implemented by a transconductance amplifier circuit (see Figure 3B). In this work, it is 2 GΩ. $\frac{v_{d\,e\,n}-v}{R_{C}}$ is the current flowing into the somatic compartment (controlled by the potential difference between dendritic and somatic compartment) *via* the unidirectional resistor. *f*_*x*_(*v*),*g*_*x*_(*v*),*andr*_*x*_(*x*) are sigmoidal functions and their equations are as follows:

(6) $$f_{x}\,\left(v\right)=\frac{M_{x}}{1+\text{exp}\,\left(-\frac{k}{U_{T}}\,\left(v-\partial_{x}\right)\right)},$$

(7) $$g_{x}\,\left(v\right)=I_{0}\,\sqrt{\frac{R_{x\,20}\cdot \text{exp}\,\left(\frac{k}{U_{T}}\,\theta_{g\,x}\right)}{1+R_{x\,21}\cdot \text{exp}\,\left(-\frac{k}{U_{T}}\,\left(v-\theta_{g\,x}\right)\right)}},$$

(8) $$r_{y}\,\left(y\right)=I_{0}\,\sqrt{\frac{\text{exp}\,\left(\frac{k}{U_{T}}\,\theta_{r\,y}\right)}{1+\text{exp}\,\left(-\frac{k}{U_{T}}\,\left(y-\theta_{r\,y}\right)\right)}},$$

where, *U_T*, *I_0*, and *k* are the thermal voltage, the current scaling parameter, and the capacitive coupling ratio of the PMOS transistor used to design the neuron circuit. Parameters ∂_*x*_, θ_gx_, and θ_ry_ control the turning point of the sigmoid function. A detailed description of this somatic compartment and its circuit implementation are found in Kohno and Aihara (2016) and Kohno et al. (2016). In the circuit, *v* is implemented as the difference between the power voltage (Vdd) and the voltage of the membrane potential node, and the polarity of excitatory and inhibitory currents is reversed. The synaptic current is depolarizing (hyperpolarizing) when it flows out (in) from the somatic compartment circuit. This circuit can reproduce six different spiking dynamics observed in biological neurons. This model has been designed from first principles (with dynamics like the Hodgkin–Huxley neuron model) and does not involve resetting of membrane potential while spiking. In the integrate-and-fire-based neuron models such as the LIF neuron model, the neuronal spike is approximated by a reset of the membrane potential. It is known that they can realize class 1 spiking dynamics in the Hodgkin’s classification (the spiking frequency of the neuron is dependent on the input current) but not class 2 dynamics (characterized by the Hopf bifurcation and bistability). An adaptive exponential integrate-and-fire (AdEx) neuron can realize class 2 spiking dynamics, but because it omits the dynamics of reproducing the neuronal spike, its phase response curve (PRC) is not generally of type 2. The spiking dynamics replicated by the somatic compartment used in this work are fast spiking (classes 1 and 2 in the Hodgkin’s classification), regular spiking, low threshold spiking, elliptic bursting, and square wave bursting, and its circuit implementation is estimated to consume less than 6 nW of static power. This versatility in spiking dynamics comes at a cost in terms of silicon area and power consumption in comparison with circuit implementation of the LIF and AdEx neuron models. An estimated static power consumption of AdEx neuron in the ROLLS chip (Qiao et al., 2015) is less than 3 nW (exact value not available). The energy consumption of a recent implementation of the AdEx neuron using lower-node FD-SOI technology has been reduced by an order of magnitude (Rubino et al., 2019).

For the task of pattern recognition here, the somatic compartment is configured to be in the fast spiking class 1 mode. In this mode, its spiking frequency increases in response to an increase in the stimulus current. The resting membrane potential *v* is set at 315 mV (in the circuit 685 mV). The dendritic compartment receives spike inputs *via* excitatory synapses whose synaptic conductance is modeled by a bi-exponential waveform mimicking AMPA-type synapses. The majority of large-scale neuromorphic implementations such as the ROLLS chip (Qiao et al., 2015) use single compartment neuron models and do not have dendritic compartments. The BrainscaleS project has implemented a multicompartment neuron model with active dendritic compartments (Schemmel et al., 2017). These dendritic compartments can generate various kinds of spikes (e.g., sodium-based action potential and calcium-based plateau potential) independent of the somatic compartment. In our implementation, we use a passive dendritic compartment with no spiking capability for simplicity of the circuitry.

The synaptic circuit implemented in this work is shown in Figure 3A. It is similar to the log-domain integrator synapse (Merolla and Boahen, 2003). The 4-bit synaptic weight is implemented using a digital to analog converter (DAC), comprising the first stage of the synaptic circuit. A brief description of the circuit operation is given next, and its details are found in Gautam and Kohno (2020). An input pulse activates the node *nV*_in_, switching on the transistor M1, and pulling its drain to *V*_*ddInt*_, and M2 along with the inverter I0 comprises a charge injection module making the transition to *V*_*ddInt*_ instantaneous. Transistors M3 to M6 are binary switches that control the value of the synaptic weight and are connected to the STDP module. Transistors M7 to M10 are matched binary-weighted transistors. The parameter voltage *sV*_w_ controls the amplitude of the synaptic current. Depending on its value and the value of the synaptic weight, the DAC stage sources a current into the node *V*_*syn*_, which is charged for the duration of the input pulse. Once the pulse turns off, the transistor M11 operating in the saturation region discharges the node *V*_*syn*_ linearly. The parameter voltage *sV*_t_ and the capacitor *C*_*syn*_ control the rate of discharge. The linear voltage *V*_*syn*_ is then converted to exponential current *via* the exponential current–voltage relationship of the MOS transistor (M12) operating in the subthreshold domain; 256 of these synaptic circuits impinge on the dendritic compartment. Because current flowing out of (into) the somatic compartment depolarizes (hyperpolarizes) the neuron, the synaptic circuit upon activation depolarizes the neuron and causes it to spike.

In the results presented in this study, a simplified synapse model that retains the bi-exponential profile of synaptic current generated by the circuit is used to model the synaptic current. It is modeled as a difference of two exponentially decaying waveforms of different time constants described by:


(9) $$I_{\text{syn}}\,\left(t\right)=I_{\text{sw}}.\left(-{\text{exp}}^{-t/\tau_{r}}+{\text{exp}}^{-t/\tau_{d}}\right)/a_{\text{scale}}$$

where, *I*_syn_(*t*) is the synaptic current. τ_*r*_ and τ_*d*_ together control the rising and falling time constant of the synaptic current, and their values are set at 1 and 3 ms, respectively. In the circuit, the time constant is controlled by *C*_*syn*_ and the voltage *sV*_t_. *a*_*scale*_ is a scaling factor so that the peak value of the synaptic current is equal to the value of the synaptic weight denoted by *I*_*sw*_. This peak occurs at around 2 ms similar to the current profile generated by the synaptic circuit. The synaptic weight of these synapses is restricted to 4 bits. The maximum amplitude of the synaptic current is fixed at 15 pA and the minimum amplitude at 0 pA. A single-bit change in the value of synaptic weight induces a change of 1 pA in the synaptic current. The synaptic current flows into the dendritic compartment described by:


(10) $$C_{d\,e\,n}\,\frac{d\,v_{d\,e\,n}}{d\,t}=I_{s\,y\,n}-\frac{v_{d\,e\,n}-E_{l\,e\,a\,k}}{R_{l\,e\,a\,k}},$$

where, *v*_*den*_ and*v* are the membrane potentials of the dendritic and somatic compartments, respectively. *C*_*den*_ is the dendritic capacitance fixed at 30 pF (for setups 1 and 2) and 12 pF (for setup 3). The value of the leak resistor *R*_*leak*_ is set at 40 MΩ for setups 1 and 2 and 80 MΩ for setup 3. The leak resistor can also be implemented using the same single-stage transconductance amplifier (Figure 3B) with the gate of transistor M4 connected to *E*_*leak*_ and the gate of transistor M5 connected to *v*_*den*_. As the number of active afferents in setup 3 is much lower, the value of leak resistance is increased and the value of membrane capacitance is reduced. *E*_*leak*_ is kept at 315 mV and it sets the membrane potential of the soma circuit at around the same value. A programmable capacitor bank along with parasitic capacitance of the synaptic circuits implements the dendritic capacitance *C*_*den*_.

## Results

We performed two groups of simulations in each setup (100 runs for each group) using the fourth-order Runge–Kutta method with a 10-μs time step. Spike pattern frequency (time intervals between repeating spike patterns) was varied between the two groups. In the first group, it was fixed at 25%, and in the second group, to make the task even more difficult, it was dropped to 10%. This increases the difficulty of the task as the neuron now comes across more random noise rather than repeating spike patterns, increasing the probability that synapses get depressed during the initial phase of the run and causes the neuron to stop spiking. The criterion for success was chosen to be a hit rate greater than 98% and zero false alarms in the last 150 s of the run similar to the criterion used in Masquelier et al. (2008).

In Table 2, results by the STDP learning with 64-bit floating-point precision and the adaptive STDP learning with 4-bit fixed-point precision are shown. In the former setup (Masquelier et al., 2008) with a pattern frequency of 25%, 96 out of 100 runs were successful and it was observed that with pattern frequency reduced to 10%, the success rate dropped to around 45% from 96%. Using the adaptive STDP learning, the performance was similar. Here (setup 1), we obtained a 94% success rate with a pattern frequency of 25%. Out of 100 runs, in four cases, the neuron stopped spiking within the first 40 s; in one case, there was a single false alarm in the last 150 s of the run (with a 100% hit rate); and in the final case of failure, the hit rate in the last 150 s was 96%. Of the successful runs, in 85 runs, the hit rate was 100%, and in 8 runs, a hit rate greater than 99% was observed. With the pattern frequency reduced to 10%, the success rate dropped to 38% in setup 1. In Table 3, the endurance of the adaptive STDP learning against the smaller number of afferents is evaluated. With 1,024 afferents (setup 2), the performance was better than even STDP learning with 64-bit floating-point precision. We obtained a 96% success rate with a pattern frequency of 25% and an 88% success rate with a pattern frequency of 10%. In the final setup with just 256 afferents, we obtained a success rate of 95% with a pattern frequency of 25% and 83% with a pattern frequency of 10%.

**TABLE 2:** Comparison of the STDP and adaptive STDP learnings.

**Performance metric**	**STDP with high-resolution synapses (** Masquelier et al., 2008 **) (%)**	**Adaptive STDP with 4-bit synapses (setup 1) (%)**
Success rate with pattern frequency of 25%	96	94
Success rate with pattern frequency of 10%	40–50	38

**TABLE 3:** Results of adaptive STDP learning for setups 2 and 3.

**Performance metric**	**Setup 2 (1,024 afferents) (%)**	**Setup 3 (256 afferents) (%)**
Success rate with pattern frequency of 25%	96	95
Success Rate with pattern frequency of 10%	88	83

The parameters controlling learning and the values of the leak resistance and the dendritic capacitance were tuned manually. They were specifically tuned for setup 1 and then used as it is for setups 2 and 3 with minor modifications. The parameters that were modified are listed in Table 4. Between setups 1 and 2, the only change made was in the initial value of synaptic weight. With the value of dendritic capacitance and leak resistor fixed, the initial value of synaptic weight determines the spiking frequency of the neuron during the initial phase of the run. The value of initial synaptic weight was set so that the spiking frequency of the neuron during the initial phase of the run stays within the range of 50 to 160 Hz. This value of initial spiking frequency is not dependent on the input spike rate or any specific task. A minimum initial firing frequency around 50 Hz makes sure that the synaptic current in the initial phase of the run is strong enough to keep the neuron spiking for learning to take place. With an initial frequency smaller than 10–15 Hz, performance starts to degrade considerably. On the higher end, it was observed that having a very high spiking frequency (>200 Hz) during the initial phase of the run led to more failures. This is intuitive because a high initial spiking frequency leads to a higher number of discharges outside the pattern leading to early depression of synapses. Also, there is a limit to the maximum spiking frequency of a biomimetic neuron model. Just like biological neurons, if the excitatory input current is too high, the neuron does not spike, and its membrane potential saturates at a value higher than its resting membrane potential.

**TABLE 4:** Parameters changed across setups.

**Parameters**	**Setup 1**	**Setup 2**	**Setup 3**
*t*_*post*_ (last step)	35.6 ms	35.6 ms	38.6 ms
Initial weight	2	3	7
*R*_*leak*_	40 MΩ	40 MΩ	80 MΩ
*C*_*den*_	30 pF	30 pF	12 pF

As the number of afferents in setup 1 doubles that of setup 2, the value of initial synaptic weight in setup 1 was chosen to be 2 (implying a synaptic current of 2 pA), and in setup 2, it was fixed at 3 (implying 3 pA). In setup 2, using an initial synaptic weight value of 3 and 4 gave similar results. However, using an initial synaptic weight of 2 did not generate sufficient synaptic current to depolarize the neuron sufficiently. In setup 3 (256 afferents), the value of the leak resistor, the dendritic capacitor, and the initial value of synaptic weight were changed. As the number of active afferents is reduced considerably, it is reasonable that the value of the leak resistor is increased, and the value of dendritic capacitance is decreased. The initial value of synaptic weight was fixed at 7 so that the initial spiking frequency is in the desired range of 50–160 Hz.

The adaptive STDP learning parameter *t*_*pre*_ was fixed at 10 ms and *t*_*post*_ was varied during learning. The first change in the value of *t*_*post*_ was made after 6 s (2 ^∗^ *t*_*adap*_) and the remaining changes were made every 3 s. The following are the values of *t*_*post*_ for each of the six steps while learning (in setups 1 and 2): 10.3, 13.3, 18.3, 23, 28.2, and 35.6 ms. The exact change in its value is not very important as long as it is not too large during the first few steps. In setup 3, the final value of *t*_*post*_ in the last step was increased to 38.6 ms instead of 35.6 ms. As the background activity in the spike train in setup 3 does not contain the additional 10 Hz noise and the spike patterns do not have any jitter, the value of *t*_*post*_ can be increased to higher values without depressing the synapses associated with the pattern. It was observed that without this change a couple of false alarms were detected in around 25% of the runs. The spike pattern hit rate is 100% in all successful runs. In setups 1 and 2, a hit rate of 100% is observed in 85 (30) and 90 (80) runs for a pattern frequency of 25% (10%), respectively.

**FIGURE 4:**
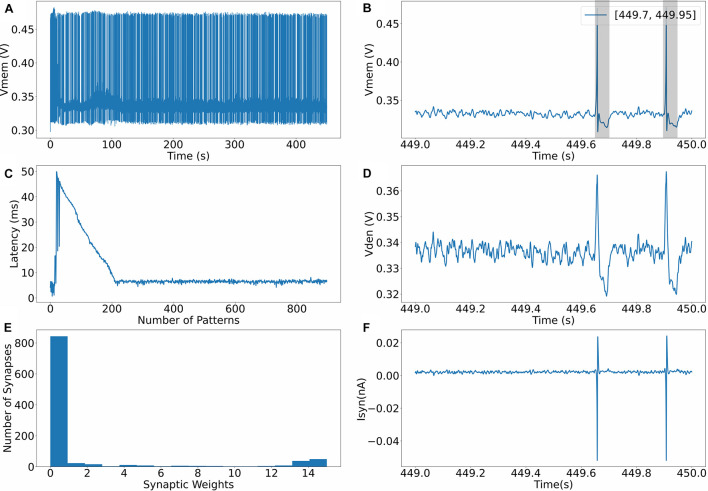
**(A)** Evolution of membrane potential during training. **(B)** Membrane potential of the somatic compartment during the last second, neuron only spikes where the pattern exists. **(C)** Latency to spike within the 50 ms long pattern. **(D)** Membrane potential of the dendritic compartment during the last second. **(E)** Bimodal distribution of synaptic weights after learning. **(F)** Synaptic current flowing into the somatic compartment via the unidirectional resistor.

The evolution of the dynamics of a neuron for one of the runs (in setup 2) is shown in Figure 4. Figure 4A shows the spiking behavior of the neuron with a pattern frequency of 10%. The spiking frequency is high during the initial phase of the run, and as the learning progresses and the neuron becomes more selective to spike inputs, the frequency decreases. Figure 4B shows the spiking behavior of the neuron in the last second; as expected, the neuron only spikes in the presence of the pattern. The times where the 50-ms pattern ends are labeled in the top right corner of the figure, and the pattern duration is marked by a box. Figure 4C shows how the time to spike within a pattern decreases while learning and settles to a value less than 10 ms. Figure 4D shows the profile of dendritic membrane potential *v*_*den*_ during the last second of the run. Figure 4E shows the bimodal distribution of synaptic weights after learning is completed, and the final figure shows the profile of synaptic current injected into the somatic compartment *via* the unidirectional resistor. The unidirectional resistor allows the current only to flow into or out of the somatic compartment, and this current is proportional to the difference between the membrane potential of the dendritic compartment and the somatic compartment. The current reverses direction during the neuronal spike when *v* > *v*_*den*_.

## Discussion and Conclusion

The presented work is built upon the spike pattern model in Masquelier et al. (2008) where the same pattern detection task was demonstrated using STDP learning. The results above show that the proposed adaptive STDP learning with 4-bit fixed-point synaptic weights is equally powerful as the STDP learning using higher resolution weights. Synaptic weight in CMOS-based synaptic circuits is generally implemented using DACs (Schemmel et al., 2010; Moradi and Indiveri, 2013) or capacitors (Azghadi et al., 2014). Both have their disadvantages. The size of these DACs increases exponentially with the number of bits needed to configure the value of synaptic weight, and the capacitors due to their physical nature have the limitation of being leaky and thus lead to depression in the value of synaptic weight over time unless an additional circuitry to refresh the charge on the capacitor is implemented. This is one of the reasons that large-scale analog neuromorphic chips like ROLLS (Qiao et al., 2015) have palimpsest (1-bit sticky) STDP synapses. However, the use of low-resolution weights leads to a degradation in performance in some tasks such as the pattern recognition discussed in this work. The proposed learning rule strives to solve this problem. Instead of increasing the weight resolution, the performance of the network is improved by adapting *t*_*post*_ (the parameter controlling depression) over the course of learning. In terms of implementation, this is advantageous because a single circuit controlling the adaptation of *t*_*post*_ can be shared with all the afferent synapses, instead of increasing the area of each synapse (when using high-resolution weights). Another simplification made in the design was to use the 1-bit rectangular STDP function (Figure 1C) instead of the exponential STDP function (Figure 1B). With the 1-bit rectangular STDP function, the bit update can be implemented by a simple 4-bit up-down counter (Mano, 2002) that saturates at the maximum (1111) and minimum (0000) count values with the state of the counter controlling the value of synaptic weight.

The performance of the network in case where pattern frequency was reduced to 10% decreases considerably in setup 1 but is relatively better in setups 2 and 3. Comparing the results in setups 1 and 2, a severe degradation in performance occurs only in setup 1. The main factor for this reduction in success rate may not be the reduced pattern frequency but the additional 1,024 afferents that do not have hidden spike patterns. A limitation of this study is that a very limited range of parameter space could be explored as all the parameters were tuned manually. Instead of using a parameter search technique like grid search, the values of parameters were decided empirically based on simulation results. The value of *t*_*adap*_ was fixed at 3 s to make sure that the synapses associated with the pattern to be detected have enough time to be potentiated (in both cases with a pattern input frequency of 25% and 10%). However, the effectiveness of the algorithm is not sensitive to *t*_*adap*_. Similar results were obtained when *t*_*adap*_was 2 and 4 s. The value of 3 s was chosen as the results were good enough across all setups to demonstrate the capability of the algorithm. The results can likely be improved further by determining the optimum value of the *t*_*adap*_ parameter either by manual tuning or some metaheuristic algorithm like differential evolution (Buhry et al., 2011). The value of *t*_*adap*_ is also independent of the input spike rate as the same value of 3 s is chosen across all simulation setups that differ in the number of active afferents and population average input spike rate (see Table 1). A larger value of *t*_*adap*_ might be needed if the spike patterns are present even more sparsely (results have been shown for 25% and 10% pattern frequency). The value of *t*_*adap*_ seems to be related to the frequency of spike patterns in the input spike train, but we have not yet worked out a systematic method to arrive at this relationship. It will be explored in future works. The values of the parameters of the dendritic compartment were modified in setup 3 (with respect to setups 1 and 2) as the number of active afferents was reduced to one-fourth of its value and their average population spike rate was smaller due to the absence of the 10-Hz noise. Though the change in the value of these circuit parameters makes intuitive sense (a smaller number of afferents imply a high value of leak resistance and low capacitance), this modification did not follow any specific scaling rule, and a proper guideline to make these modifications will also be explored in future works.

In a pattern detection task like the one described in this study, upon completion of learning, the STDP rule potentiates the weight on synapses that are activated near the beginning of the pattern. That is, during the learning phase, the time to spike within a pattern decreases, and upon completion of learning, the neuron spikes near the beginning of the pattern. While learning with each pattern presentation, the neuron initially spikes at a random time within the 50-ms pattern. The STDP learning then potentiates the weight of the synapses that fired just before the spike of the postsynaptic neuron; due to this, during the next pattern presentation, the neuron spikes a bit earlier. For the spike latency to decrease continuously, the spike density of the input must be high enough (throughout the 50-ms duration of the pattern) for a group of synapses to be activated in close temporal proximity to one another and cause the neuron to spike. Though we could not arrive at a specific minimum value necessary, this is more likely to happen if the number of afferents is high. In our simulation setups, we observed this latency in 93 runs in setup 1 and 87 runs in setup 2 with a pattern frequency of 25%. In these cases, the neuron spiked within 10 ms of the pattern presentation after learning. However, in setup 3, this reduction in spike latency was not observed in most of the cases because the number of afferents is too small leading to a sparser input spike density. The neuron becomes sensitive to a part of the pattern and spikes repeatedly at the same part with negligible change in timing of the spike in subsequent pattern presentations.

A trade-off in the performance must exist between the number of neurons used to detect patterns and the resolution of synaptic weights, but this trade-off was not explored in this study. Increasing the number of neurons should theoretically improve the performance, but it is likely that there is some minimum limit below which the value of synaptic efficacy cannot be reduced if high performance is desired.

The neuron model used in this study was a two-compartment model with a unidirectional resistor. This model was chosen because of its ease of implementation. It can be implemented using a transconductance circuit that consumes very low static power in the picowatts range. However, the use of the proposed adaptive STDP learning is not restricted to two-compartment models and can be used with single compartment point neuron models, too. We verified this using setup 3 and obtained a success rate of 85% with a pattern frequency of 25% using the same biomimetic somatic compartment (with a completely different set of parameters). The somatic compartment (Kohno and Aihara, 2016) was configured to operate in fast spiking class 1 mode. As the natural spiking mode of a bioinspired LIF neuron is similar to the fast spiking class 1 mode (where the spiking frequency of the neuron increases with increased stimulus current), the proposed learning rule may be applicable with a single compartment LIF neuron, like in the study of Masquelier et al. (2008).

The primary motivation behind the proposed adaptive STDP learning is to reduce the circuit footprint, synaptic weight resolution, and complexity of the learning circuit without degrading its performance. This is done at two levels: first, the learning rule does not require the use of very high-resolution weights, and second, the change in the value of synaptic weight is restricted to a single bit for a learning step. Relatively lower resolution weights such as the 4-bit fixed point used in this study consume relatively less silicon area and power, and restricting the change in the value of synaptic weight to a single bit simplifies the design of STDP circuits. Based on simulation results, we showed that the proposed adaptive STDP learning is equally powerful as conventional high-precision STDP learning. We reported how the network dynamics evolve with variation in the value of *t*_*post*_ alone. Changing the value of *t*_*pre*_ might have other interesting effects and will be explored in the future. It is also possible that such simple adaptation mechanisms are taking place in the brain. This idea of adapting the value of parameters controlling depression can also be extended to the STDP learning with high-resolution synaptic weight. One of our next steps is to verify the capability of the proposed adaptive STDP rule in a more biologically realistic and competitive setting with multiple neurons tasked with detecting multiple spike patterns hidden in the input spike train like the study presented in Masquelier et al. (2009).

The simulation results discussed in this study are demonstrated using ideal circuit models in python instead of a circuit simulator. This was done because of the prohibitively high computational resources needed to simulate the network with transistor-level schematics. The potential impact of circuit mismatch and thermal noise on the performance of the adaptive STDP learning was not verified by simulation and will be evaluated in the future, also on the fabricated chip. The mismatch will cause significant variation in the values of the learning parameters *t*_*pre*_ and *t*_*post*_ along with the maximum amplitude and time constant of synaptic current for each synapse. However, as the proposed adaptive STDP is a local learning rule and the performance of the proposed rule is not dependent on the precise values of the learning parameters, we intuitively expect the that impact of mismatch between synapses will not be too drastic, and the adaptation of the parameter *t*_*post*_ might also ameliorate the negative impact of mismatch on learning if any. The unidirectional two-compartment neuron with 256 afferents discussed in this study has been submitted for fabrication in the Taiwan Manufacturing Semiconductor Company (TSMC) 250 nm technology node. In the Spectre circuit simulator, the static power consumption of a single synapse and the energy per spike (to generate a synaptic current of amplitude 15 pA and time constant 3 ms) are less than 2 pW and 200 fJ, respectively. The static power consumption of the learning circuit is less than 135 pW, and the energy to process a pair of pre- and postsynaptic spikes corresponding to a single learning step is less than 235 pJ. A single 4-bit synapse (capable of being configured as excitatory, inhibitory, or shunting inhibitory) along with its learning circuitry and digital memory occupies around 17,250 m^2^of silicon area. Its experimental results will be reported in the near future. The implementation is not limited to any technology node; 250 nm was used because of its availability including financial considerations. The proposed adaptive STDP rule can be implemented in fine lower technology nodes. We plan to move to 28 nm FD-SOI in the future.

## Data Availability Statement

The original contributions presented in the study are included in the article/supplementary material, further inquiries can be directed to the corresponding author.

## Author Contributions

AG performed the study. TK supervised it. Both authors contributed to the article and approved the submitted version.

## Conflict of Interest

The authors declare that the research was conducted in the absence of any commercial or financial relationships that could be construed as a potential conflict of interest.

## Publisher’s Note

All claims expressed in this article are solely those of the authors and do not necessarily represent those of their affiliated organizations, or those of the publisher, the editors and the reviewers. Any product that may be evaluated in this article, or claim that may be made by its manufacturer, is not guaranteed or endorsed by the publisher.
